# Vertical bearing capacity of a pile-liquefiable sandy soil foundation under horizontal seismic force

**DOI:** 10.1371/journal.pone.0229532

**Published:** 2020-03-19

**Authors:** Huang Zhanfang, Bai Xiaohong, Yin Chao, Wang Yanping

**Affiliations:** 1 School of Civil and Architecture Engineering, Shandong University of Technology, Zibo, China; 2 College of Civil Engineering, Taiyuan University of Technology, Taiyuan, China; China University of Mining and Technology, CHINA

## Abstract

In this study, research on a pile group system was conducted using shaking table tests under four working conditions: a natural foundation and pile-spacing conditions of 3D, 3.5D, and 4D (D is the diameter of the pile). The time histories of the excess pore pressure ratio and settlement were analyzed. It was determined that pile foundations improved the anti-liquefaction performance of the soil, and the effect was much greater when the pile spacing was 3D. In addition, the settlement dynamic amplification factor (SDAF) was proposed and calculated at different vibration times. The result was fitted with a linear relationship, and the correlation coefficient was relatively high. During the aseismic design of the pile foundation bearing capacity, the SDAF was multiplied by the static load, and the results showed that in the dynamic design of the pile foundation, the dynamic problem can be transformed into a static analysis, which can provide a reference for the design of the vertical bearing capacity of pile foundations under seismic force.

## Introduction

A pile foundation is a type of deep foundation that is widely used due to its large bearing capacity, low distortion and high reliability. However, recent large earthquakes indicate that the loss of life and property caused by damage to the pile foundation is serious, and the main cause is the reduction in the vertical and horizontal bearing capacities. Scholars have made some achievements in the research of the dynamic characteristics of pile foundations.

Scholars have studied related topics by means of centrifugal model tests. Scott[1] used a centrifuge to carry out a horizontal cyclic load test of piles in sand and determined the basic relationship between the soil reaction and damping. Kutter B L.[2] carried out dynamic centrifugal tests on horizontally loaded piles in saturated, medium-dense sand and determined that the vibration frequency and pile-soil gap are the main factors influencing the dynamic p-y curves of pile foundations. Chacko[3] calculated and analyzed the response of a pile foundation under a vibration load according to the centrifugal model test of a single pile in saturated sand and determined the accuracy of their analysis of the pile-soil interactions. These authors found that the accuracy of their analysis depended on the accuracy of the free field motion calculation. Liu, Dobry, Abdoun, Wilson, et al.[4], using a centrifugal model test, performed considerable research on the horizontal bearing capacity of pile foundations in liquefied sand. These authors proposed a method to determine the p-y curve of the pile-soil interactions in a liquefied soil layer using the reduction coefficient method and determined the reduction coefficients of soil resistance after the liquefaction of varying densities of saturated sand.

Some scholars have conducted relevant research with the help of shaking table model tests. LI et al.[5] studied the lateral dynamic response characteristics of a vertical pile-group cap in nonliquefied, saturated sand with thicknesses of 300 mm and 380 mm using a shaking table test. In 1969, Kubo[6] studied the mechanical characteristics of modeled piles in sand under varying frequency sine wave excitations based on the shaking table test. However, due to the limited test conditions at that time, the effects of the model similarity ratio and boundary conditions were not considered. In 1984, Mizuno and Iiba[7] conducted a shaking table test of the pile-soil interactions using seismic wave excitation. Subsequently, many scholars began to focus on the effects of the model similarity ratio and boundary conditions on the test results but only compared their results with the prototypical structure analysis. In 1987, to prevent vibration wave reflection, Gohl and Finn[8] designed a rigid container with two inner sides to support styrene foam plastic in a shaking table model test of the pile-soil interactions. It was concluded that the vibration frequency of a pile foundation decreases due to the weakening of the soil surrounding the pile under the action of a vibration load. In 1988, Stanton et al.[9] made a cylindrical flexible vessel to carry out shaking table tests of model piles in dry sand. The general trends in the static and dynamic responses of the pile foundations were obtained, and the test results were in good agreement with the theoretical results. Since then, the shaking table test has gradually evolved, focusing not only on the similarity ratio of the test device and model but also on comparisons of the test and theoretical results to test the rationality of the selection of the soil parameters in the theoretical analysis. In 1991, Nomura et al.[10] used a layered shear deformation box to analyze the response of pile foundations with varying rigidities in saturated sand, combining the effective stress analysis method of the free field seismic response with the horizontal reaction model of the foundation. The simulation results were calculated according to the test conditions. The calculated results were highly consistent with the test results. In 1997, Makris et al.[11] carried out a shaking table test on a single pile in dry sand and compared the test results with those calculated by the Winkler foundation beam model. The results of the test and model were highly consistent. Tamura, Minowa, Tokimastu, Fujii, et al.[12][13] used a shear box to conduct a large-scale shaking table test of the pile-soil interaction to determine the horizontal bearing characteristics of piles in liquefied soil under vibration load. The results showed that the soil stress was mainly caused by the inertial force of the superstructure before liquefaction, while after liquefaction, the soil stress was mainly due to the deformation of the soil. Additionally, before liquefaction, the p-y relationship was linear, while after liquefaction, with increasing pore water pressure and the effect of the horizontal cyclic load, the relationship gradually became nonlinear. In addition, in 2004, Wang and Feng [14] used a shaking table test to explore the relationship between the attenuation of the p-y curve parameters and the cumulative pore pressure ratio of the soil layer. However, due to the rapid increase in the accumulated pore pressure in the saturated sand under a vibration load, it was difficult to determine the quantitative relationship between the attenuation of the p-y curve of the pile-soil interaction and the cumulative pore pressure ratio of the soil layer in the process of liquefaction.

With the rise of numerical analysis, some scholars have used the finite element method to carry out correlation analysis. Jiang [15] analyzed the horizontal, rocking and horizontal-rocking dynamic response of pile groups in liquefied soil using the principle of superposition based on dynamic interaction factors. Huang [16] calculated and analyzed structure modes with different liquefaction degrees and overburdens. The differences in the results between the m-method and P-Y method were compared. Li [17] established a finite element model that considered the soil-pile-bridge interaction but did not consider the pile-soil interaction using Abaqus finite element software. Based on the principle of effective stress, Qi [18] proposed a model test method to simulate the vibrational interaction between saturated sand with residual pore pressure and the pile. The p-y relationship of the pile-soil interaction in the weakened saturated sand layer was analyzed, and the variation in the necessary parameters for determining the p-y curve of the weakened soil layer was studied. Many scholars have performed relevant full-scale experiments. Xin [19] established a simplified numerical model based on Winkler theory and combined with a new p-y curve, which considered the effect of mass in the vicinity of the pile and pore pressure on the dynamic response and stiffness of sand, to research pile-soil-bridge structure dynamic interaction in liquefying ground. Zhang et al. [20][21][22] proposed and verified a calculation method for studying the interaction between structures and soil under dynamic loads.

In addition, some scholars have completed full-scale experiments on this topic. In 1987, Ting[23] carried out a full-scale single pile test in saturated sand and determined that the dynamic p-y curve of a pile in shallow soil exhibited strain softening and hysteresis and was influenced by the pile-soil gap. Brown et al.[24][25] also discussed the pile group effect in sand using field horizontal cyclic load tests of full-scale piles. In 1989, Han et al.[26][27][28] studied the p-y curve of a single pile under a horizontal dynamic load using the horizontal excitation test of a steel pipe pile in sandy soil. Discussing the factors influencing pile-soil interaction under a vibration load, the authors determined that the p-y curve of a pile under a cyclic load was related to the vibration frequency, damping, and separation clearance of the pile-soil interface. Li [29] proposed a modified model of the dynamic p-y curves of a pile foundation in a liquefiable soil layer, and its reliability was also verified.

In conclusion, there are many methods for studying the pile-soil dynamic response. The shaking table test is an effective method, and the pile-soil interaction shaking table test has been used widely; however, most research has been based on the horizontal bearing properties of the pile foundation under a horizontal earthquake, and less research has been based on the vertical load-bearing characteristics. As a result, in this paper, the vertical load-bearing characteristics were studied using the shaking table test. The main research contents are as follows:

1. The excess pore water pressures in the soil layer were analyzed under four working conditions: natural foundation, 3D, 3.5D, and 4D (D is the diameter of the pile) pile spacing conditions.
2. The vibration settlement time history was analyzed under different working conditions.
3. The settlement dynamic amplification factor (SDAF) was proposed and calculated at different vibration times.

## 1. The experiment

### 1.1 Test system

**Fig 1:**
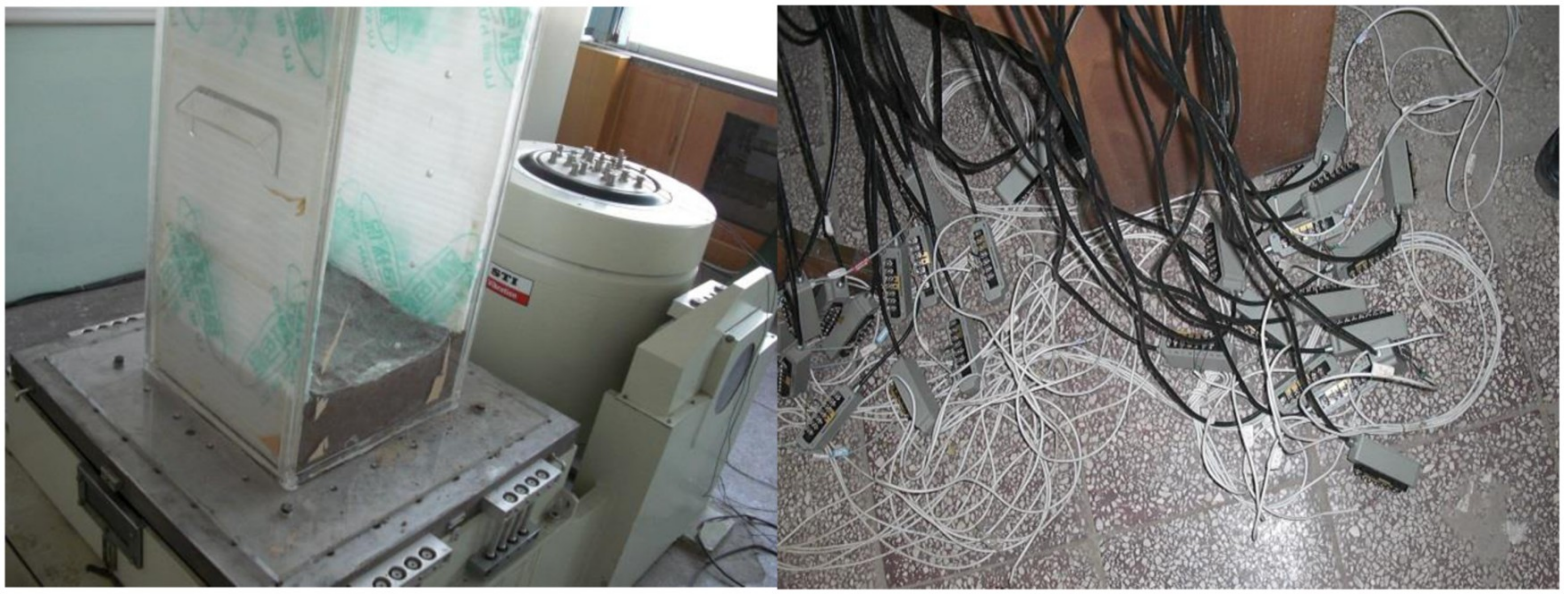
Test system.

The test system is composed of three parts: the power supply device, vibration table and control system. The power supply provides the horizontal and vertical vibration force. The rated exciting force is 21.56 kN, the rated acceleration is 980 m/s^2^, the rated displacement is 51 mm, the rated frequency range is 5–3000 Hz, and the maximum load is 300 kg. The test system is attached to a DH5922/23 data acquisition system, as shown in Fig 1.

### 1.2 Model box

**Fig 2:**
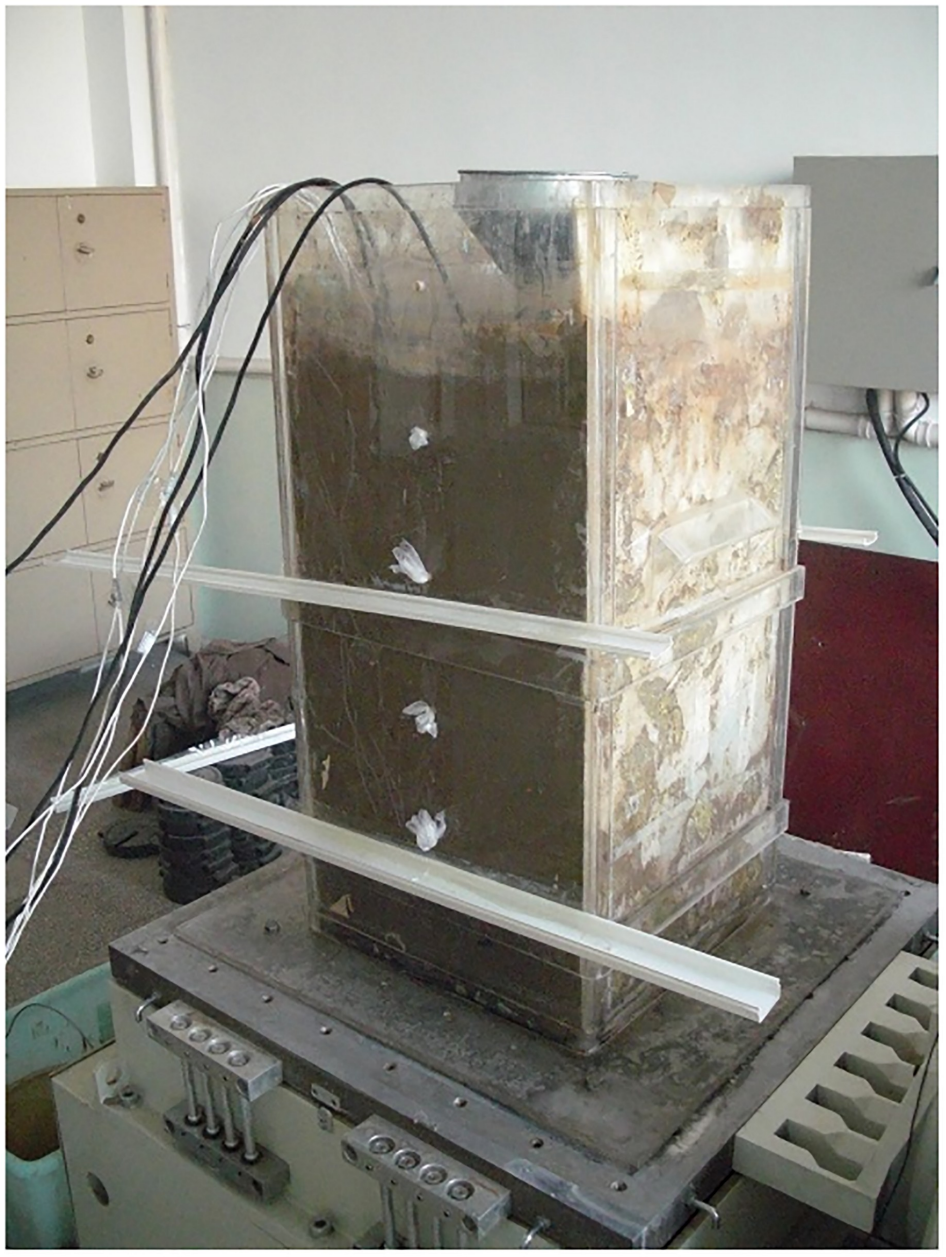
Model box.

The model box is made of organic glass with a 400 mm × 400 mm × 900 mm cubic form and 10 mm thickness. The surface is bonded with chloroform, and each side of the base plate extends 150 mm and is fixed to the vibration table. The opposite sides have a 5 mm-diameter hole along the bottom edge at intervals of 150 mm to ensure drainage. A 10 mm-thick polyethylene plate is adhered to the wall on both sides of the vibration direction, and the boundary condition of the free space is simulated. The epoxy resin is bonded to the bottom surface of the box to increase the friction force between the soil body and bottom plate, as shown in Fig 2.

### 1.3 Model pile and pile cap

A series of preliminary experiments of the concrete model pile was performed according to relevant dynamic similarity theory. The material mass ratio of the model pile is cement: sand: soil water = 1:5.8:1.45:1.9. The acceleration ratio is the ratio of *a*_*m*_ to *a*_*p*_, namely, $s_{a}=\frac{a_{m}}{a_{p}}=1.86$ (*a*_*m*_ denotes the model acceleration and *a*_*p*_ denotes the prototype acceleration). A precast pile was made, and a PVC pipe 600 mm in length and 30 mm in diameter with an outer wall thickness of 1 mm was used as a mold. The parameters of the prototype pile and model pile are shown in Table 1.

**Table 1:** Main parameters of pile.

Parameter	Model	Prototype
Pile Diameter (cm)	3	30
Pile Length (cm)	60	600
Density (kg/ m^3^)	1.766×10^3^	2.4×10^3^
Elastic Modulus (MPa)	0.41×10^4^	3.0×10^4^

The cap material was a steel plate with a thickness of 30 mm and an area of 300 × 300 mm^2^. The plate had a blind hole at a depth of 20 mm. Before the test, the precast pile was inserted into the corresponding hole, and the pile and cap were fixed.

The model soil contains two parts: the upper soil is saturated sand, and the bearing layer soil is sticky soil. The saturated sand was prepared by the dry loading method according to the calculated control parameters, and the sand was put into the model box. After preparation, the water from the top was injected into the sand to saturation. Drainage consolidation of the saturated sand was completed with the aid of a 5 mm-diameter hole reserved on the sidewall of the model box. The grain composition of the sand sample is shown in Table 2. The related parameters of the clay and sand samples are shown in Table 3.

**Table 2:** Grain composition of sand sample.

Particle Size (mm)	>1.25	1.25~0.63	0.63~0.3
Percentage Content (%)	1.81	1.1	13.75
Particle Size (mm)	0.3~0.16	0.16~0.1	<0.1
Percentage Content (%)	48.61	10.02	24.71

**Table 3:** Related parameters of clay and sand sample.

Sticky Soil	Water Content (%)	Cohesive Force (kPa)	Internal Friction Angle (°)	Permeability Coefficient (m/s)	Density (g/cm^3^)
	10	12.8	16	1×10^−5^	1.43
Sand	Maximum Dry Density (g/cm^3^)	Minimum Dry Density (g/cm^3^)	Inhomogeneity Coefficient	Saturation	Soil Grain Density (g/cm^3^)
	1.798	1.201	3.75	90	1.88

### 1.5 Test plan

**Fig 3:**
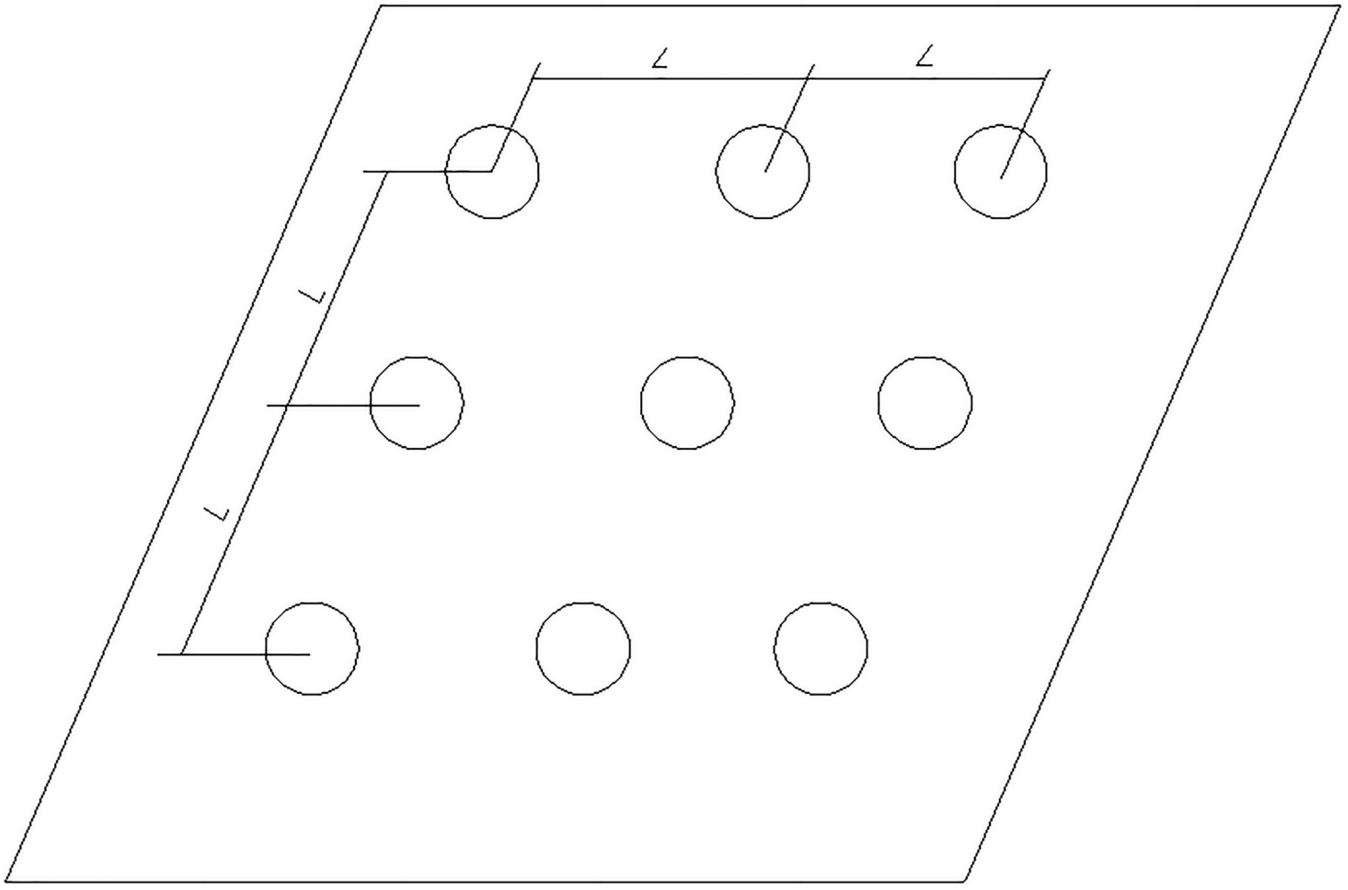
Plan location of pile group.

The location of the pile group is shown in Fig 3. The test contains four working conditions: the natural foundation and the pile group foundation with pile spacings of 3D, 3.5D, and 4D (D is the diameter of the pile).

**Fig 4:**
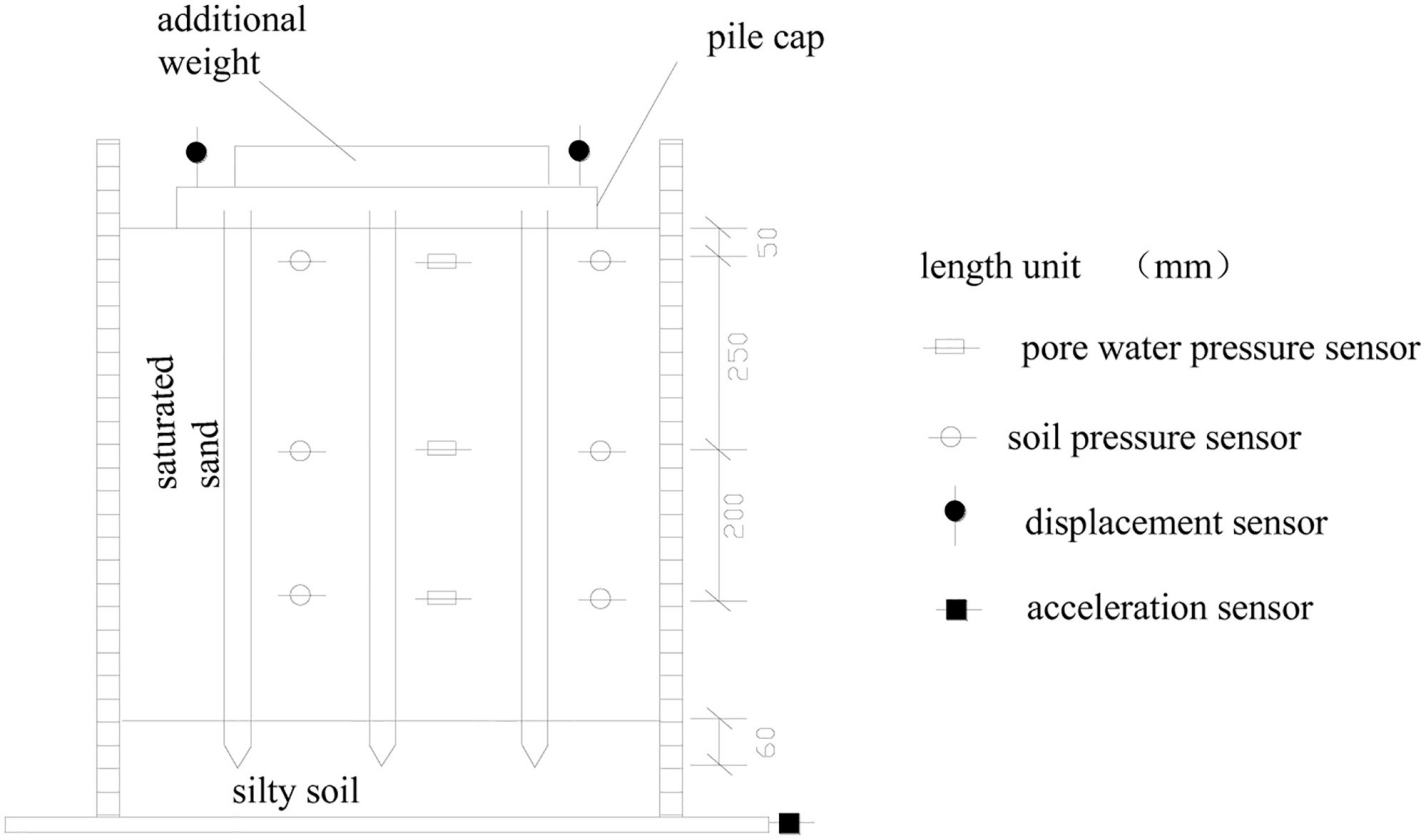
Layout of test instrumentation for pile group.

The sensor arrangement of the test is shown in Fig 4. The pore water pressure was measured using a type of DYS-3 resistance strain osmometer. The soil pressure sensor was a type of DZ-1 resistance strain sensor with a measuring range of 0.4 MPa. The vertical settlement of the pile foundation was measured in the four corners of the pile cap. The parameters of the earth pressure cells and pore water pressure sensor are shown in Tables 4 and 5.

**Table 4:** Parameters of earth pressure cells.

Type	Range (MPa)	Specifications (mm)	Sensor line length (m)
DZ-I	0.4	Φ17×7	4
DZ-I	0.1	Φ17×7	4

**Table 5:** Parameters of pore water pressure sensor.

Type	Number	Range (MP_a_)	Resolution ratio	Synthetic Error	Specifications (mm)
DYS-3	262	0.07	0.114%F·S	0.641%F·S	Φ19×30
DYS-3	183	0.07	0.159%F·S	0.721%F·S	Φ19×30
DYS-3	198	0.07	0.125%F·S	0.997%F·S	Φ19×30
DYS-3	187	0.07	0.125%F·S	0.784%F·S	Φ19×30

**Fig 5:**
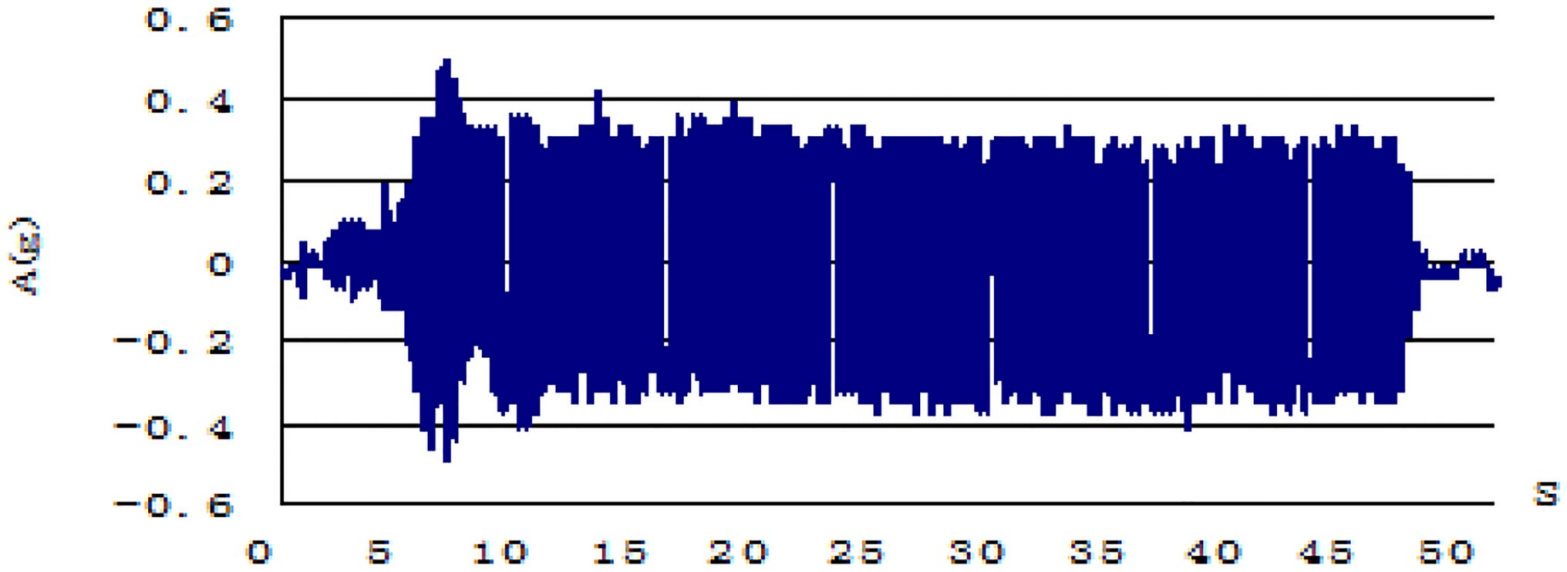
Acceleration versus time date.

The shaking table test simulates the actual working conditions: a basic earthquake acceleration of 0.2 g and an anti-seismic fortification intensity of 8 degrees. According to the dynamic similarity theory of the test model, the test input acceleration of 0.372 g was obtained by the dynamic similarity ratio calculation, the output vibration frequency was 4.313 Hz, and the input signal was a sine wave signal. The total vibration time was 48 s. The acceleration waveform of the vibration table top is shown in Fig 5.

## 2 Test results and analysis

### 2.1 Excess pore water pressure in the soil layer

The pore water pressure ratio (the ratio of excess pore water pressure to initial effective stress) is an ideal parameter to reflect the degree of soil liquefaction. In the experiment, the excess pore water pressure could be measured indirectly using the resistance strain osmometer, and the initial effective stress could be determined in two ways. One way was to test; however, accuracy was low due to various factors. The other way was theoretical calculation, namely, the calculation of effective geostatic stress and additional stress. However, to calculate the additional stress, we needed to know the load allocation between the pile and soil in the group pile foundation; the load ratio of the pile to soil was uncertain under different conditions. Through synthetic comparison, the initial effective stress was determined using the theoretical calculation results, and the value of the measured point of the soil was calculated by the load sharing ratio proposed in the literature [30].

The initial effective stress of the corresponding measuring points is shown in Table 6 according to the calculations of the soil pressure sensor.

**Table 6:** The initial effective stress of the soil at different depths under different working conditions.

Working Condition Distance From Soil Surface	Natural Foundation	Pile Foundation with 3D Pile Spacing	Pile Foundation with 3.5D Pile Spacing	Pile Foundation with 4D Pile Spacing
5cm	0.339	0.981	0.981	0.981
30cm	2.036	2.677	2.677	2.677
50cm	3.393	4.034	4.034	4.034

The unit of initial effective stress is kPa.

**Fig 6:**
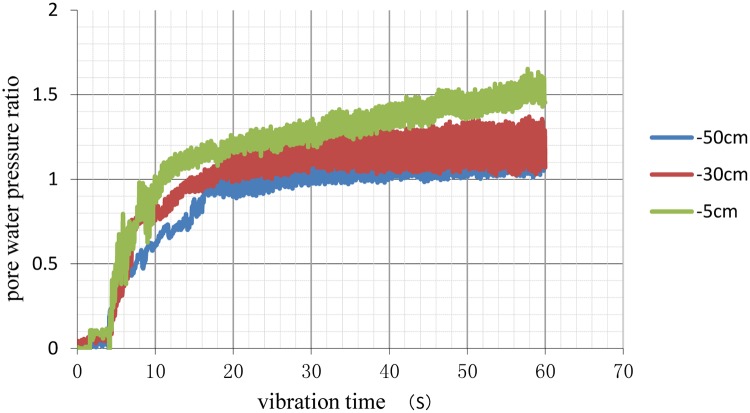
Time history of excess pore pressure ratios at different depths of natural foundation.

**Fig 7:**
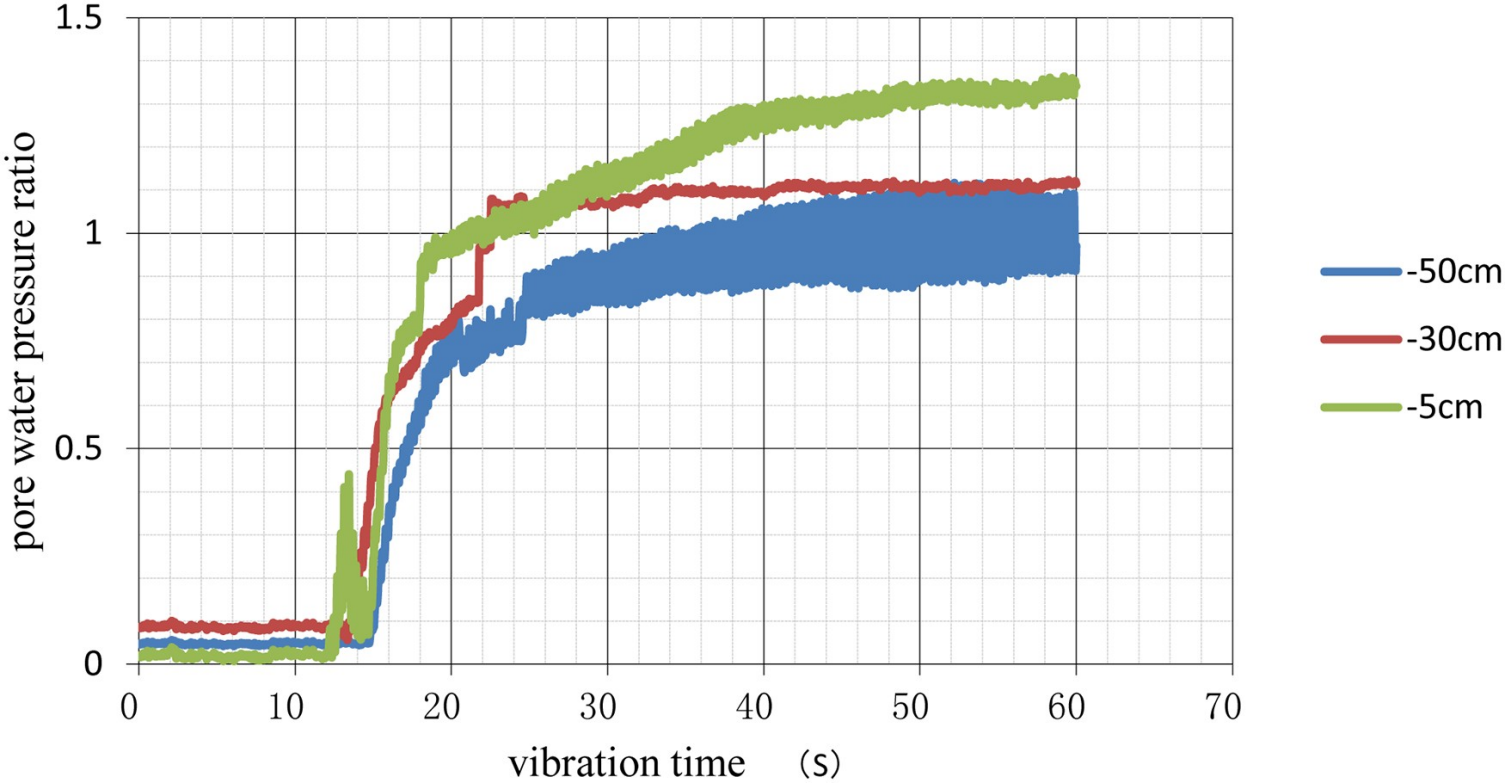
Time history of excess pore pressure ratios at different depths under 3D spacing conditions.

**Fig 8:**
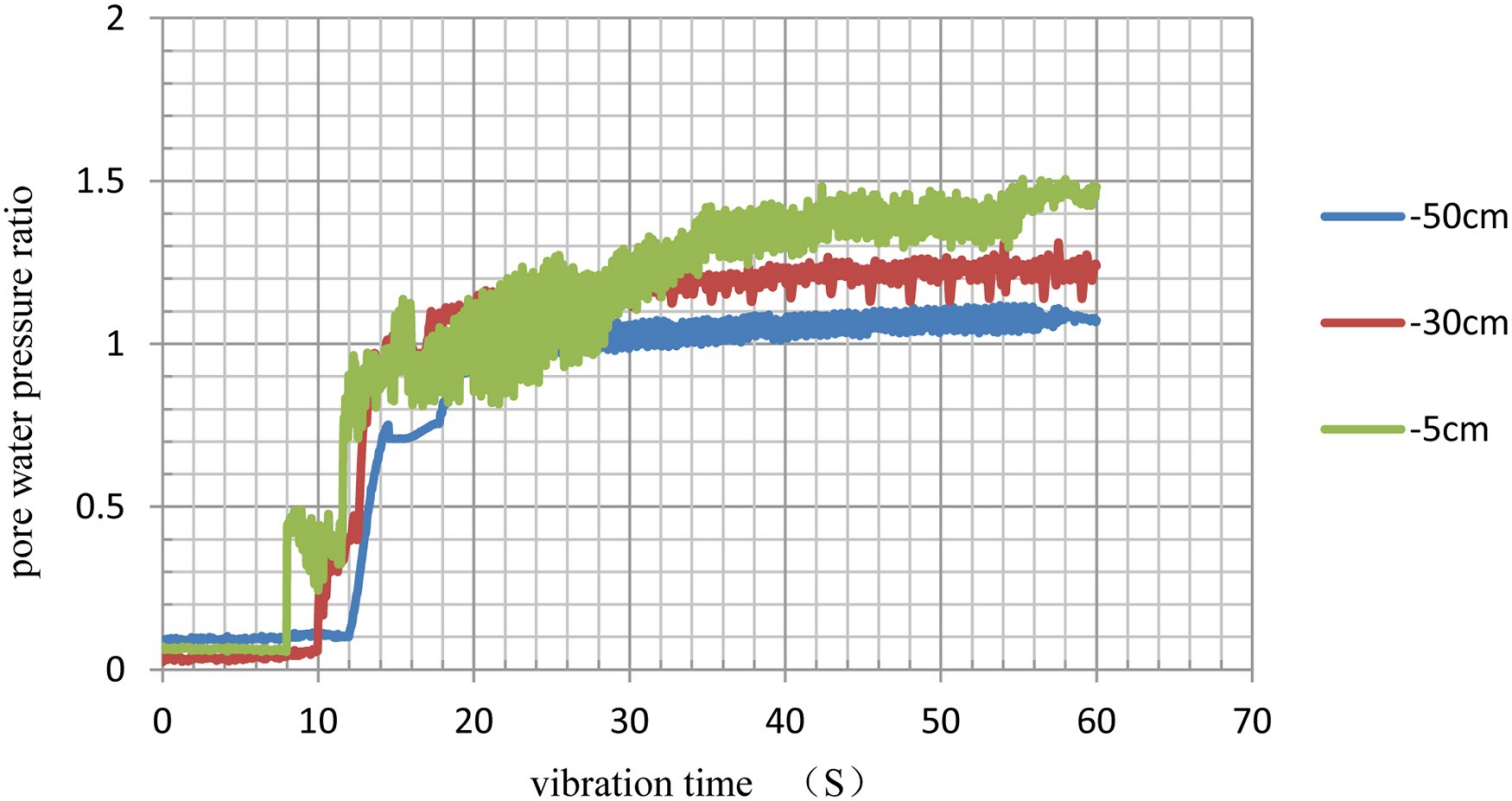
Time history of excess pore pressure ratios at different depths under 3.5D spacing conditions.

**Fig 9:**
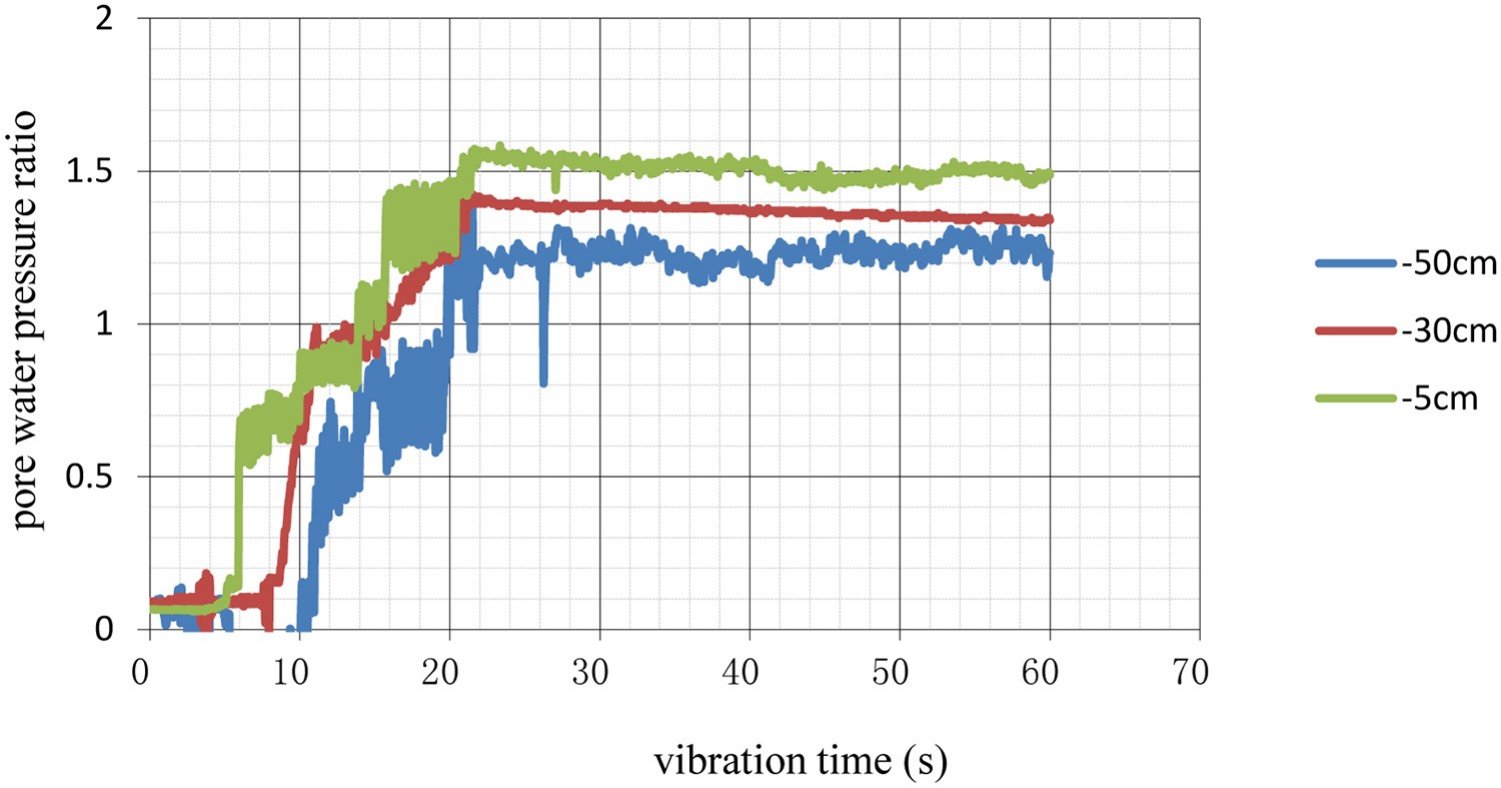
Time history of excess pore pressure ratios at different depths under 4D spacing conditions.

The curves of the pore pressure under different conditions were calculated and plotted as shown in Figs 6–9.

When the pore water pressure ratio reached 0.8, the soil began to liquefy. The results of the analysis shown in Table 7, including times when excess pore water pressure occurred and soil liquefaction began.

**Table 7:** Time of excess pore water pressure appearance and the time of soil liquefaction unit: S.

	Natural Foundation	Pile Foundation with 3D Pile Spacing	Pile Foundation with 3.5D Pile Spacing	Pile Foundation with 4D Pile Spacing
5cm	4/8	12/18	8/12	6/10
30cm	5/11	13/20	10/13	8/12
50cm	5/15	15/25	12/18	11/16

The numbers before '/' in Table 5 indicate the time when excess pore water pressure occurred, and the numbers after '/' indicate the time when liquefaction began.

As shown in Table 7, the times when excess pore water pressure occurred and pile foundation liquefaction began were later than those for the natural foundation at the same depth. These findings effectively improved the anti-liquefaction performance of the soils. Comparing the different pile spacing conditions, the time to liquefaction of the 3D pile spacing was the longest, followed, in decreasing order, by that of the 3.5D and 4D spacings. Therefore, in liquefied soil, pile foundations with a 3D pile spacing can improve the anti-liquefaction performance of the soil, providing a reference for the design of pile foundations in a liquefied field.

### 2.2 Vibration settlement time history analysis

For dynamic model tests, step-loading is difficult to accomplish in the course of vibration. Therefore, the vertical bearing capacity of the pile foundation during the vibration process was indirectly analyzed with the aid of the time history of the settlement of the pile foundation under a certain load.

**Fig 10:**
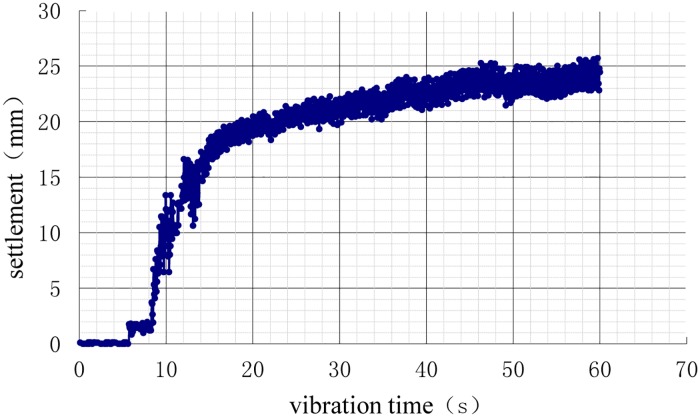
Time history of settlements during vibration of the natural foundation.

**Fig 11:**
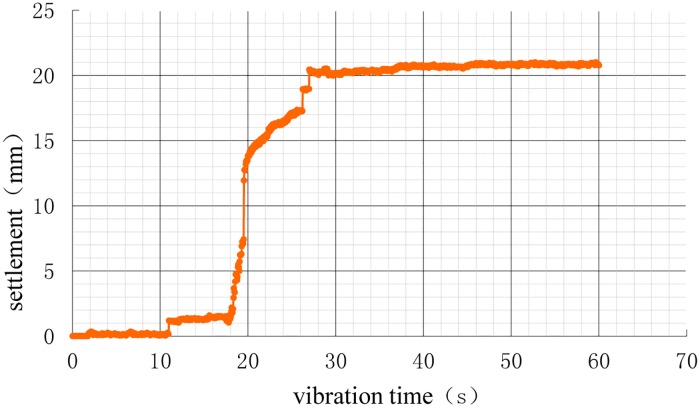
Time history of settlement during vibration under 3D working conditions.

**Fig 12:**
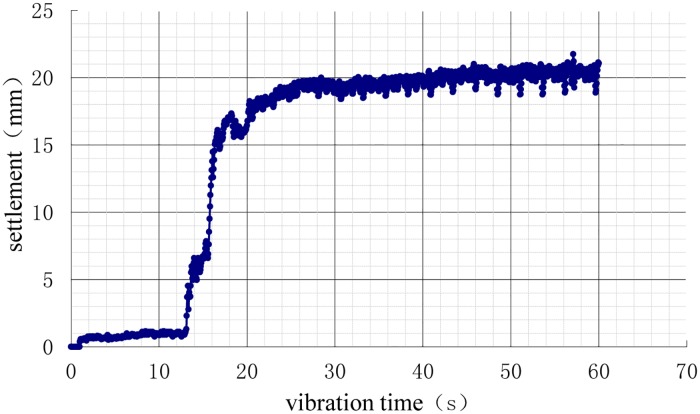
Time history of settlements during vibration under 3.5D working conditions.

**Fig 13:**
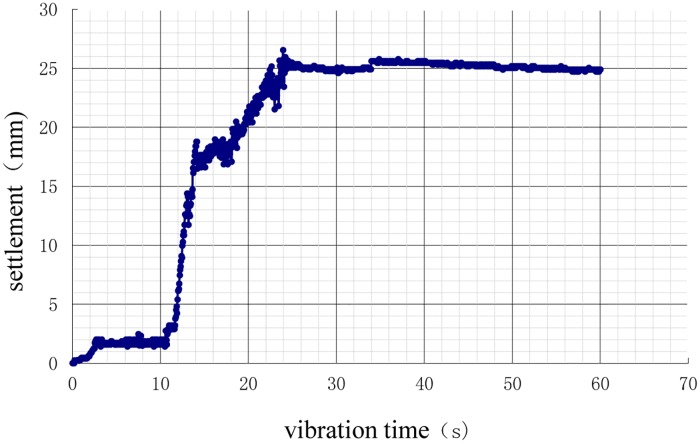
Time history of settlement during vibration under 4D working conditions.

After the model of each working condition was prepared, the displacement sensors were placed on the pile cap, and a 750 N weight was applied to the pile cap for 24 h. The settlements of the vibration process (i.e., additional settlement) are shown in Figs 10–13.

As shown in the settlement curves in Figs 10–13, there was virtually no vibration settlement in the first few seconds; a smaller settlement occurred over the next few seconds, and a small settlement was maintained over the next few seconds, followed by a sudden large settlement. A statistical summary of the times of the sudden change in settlement and the maximum settlements under the different conditions is shown in Table 8.

**Table 8:** Maximum settlement and the time of large settlement occurrence.

	Natural Foundation	Pile Foundation with 3D Pile Spacing	Pile Foundation with 3.5D Pile Spacing	Pile Foundation with 4D Pile Spacing
Time at Large Settlement (s)	8	18.5	13	10.8
Maximum Settlement (mm)	25	21.5	22	25

The maximum settlement of the natural foundation was the largest. If the additional load had been applied to the natural foundation, the maximum settlement of the natural foundation would have been greater. The maximum settlement of the pile foundation was lower, and the 3D condition was the least, followed, in increasing order of maximum settlement, by 3.5D and 4D. This result showed that the vertical bearing performance of the 3D condition was the best, demonstrating that pile foundations can improve the liquefaction resistance of the interpile soil and delay soil liquefaction. This observation was consistent with the results of the analysis of the pore pressure ratio. According to the comparative analyses in Tables 5 and 6, the larger settlement occurred in the first few seconds after soil liquefaction. The liquefaction of the soil around the pile reduced the vertical bearing capacity of the pile foundation (reduction in pile side friction resistance) such that the settlement rate increased.

The vertical bearing performance of the pile foundation system during vibration was studied directly using dynamic analysis or indirectly using static analysis with the contribution of the load dynamic amplification factor (LDAF). The dynamic performance was correlated with the vibration time. As shown in Figs 10–13, the settlements were correlated with vibration time, as the size of the settlement was closely related to the load, requiring the study of the settlement dynamic amplification factor (SDAF) instead of the LDAF, expressed as:

$$\text{SDAF}=\frac{\text{the}\;\text{total}\;\text{settlement}\;\text{of}\;\text{pile}\;\text{foundation}\;\text{at}\;\text{different}\;\text{vibration}\;\text{time}}{\text{the}\;\text{settlement}\;\text{before}\;\text{vibration}\;\text{under}\;\text{the}\;\text{same}\;\text{load}}$$

For each working condition, within 10–30 s of vibration, the settlements changed substantially, and the settlements at 15 s, 20 s, 25 s, and 30 s vibration were analyzed. Table 9 lists the pile foundation settlements at vibration times of 15 s, 20 s, and 25 s under the different conditions.

**Table 9:** Settlement at different vibrating moments.

Pile Spacing	Settlement When Static Load is 750N (mm)	Settlement of Different Vibration Time (mm)			
		15 s	20 s	25 s	30 s
3D	3.6	1.5 (5.1)	13 (16.6)	17 (20.6)	20.1 (23.7)
3.5D	3.5	6 (9.5)	17 (20.5)	19 (22.5)	21.1 (24.6)
4D	2.5	18 (20.5)	21 (23.5)	25 (27.5)	25 (27.5)

The total settlement under dynamic load is shown in brackets in the table. The total settlement at a certain moment is the sum of the vibration settlement at the moment and the settlement before vibration.

The SDAFs at different vibration times and for different pile spacings are shown in Table 10.

**Table 10:** The SDAF at different vibrating moments.

Pile Spacing	SDAF of Settlement of Different Vibration Time			
	15 s	20 s	25 s	30 s
3D	1.42	4.62	5.73	6.59
3.5D	2.72	5.86	6.43	7.03
4D	8.20	9.40	11.00	11.00

**Fig 14:**
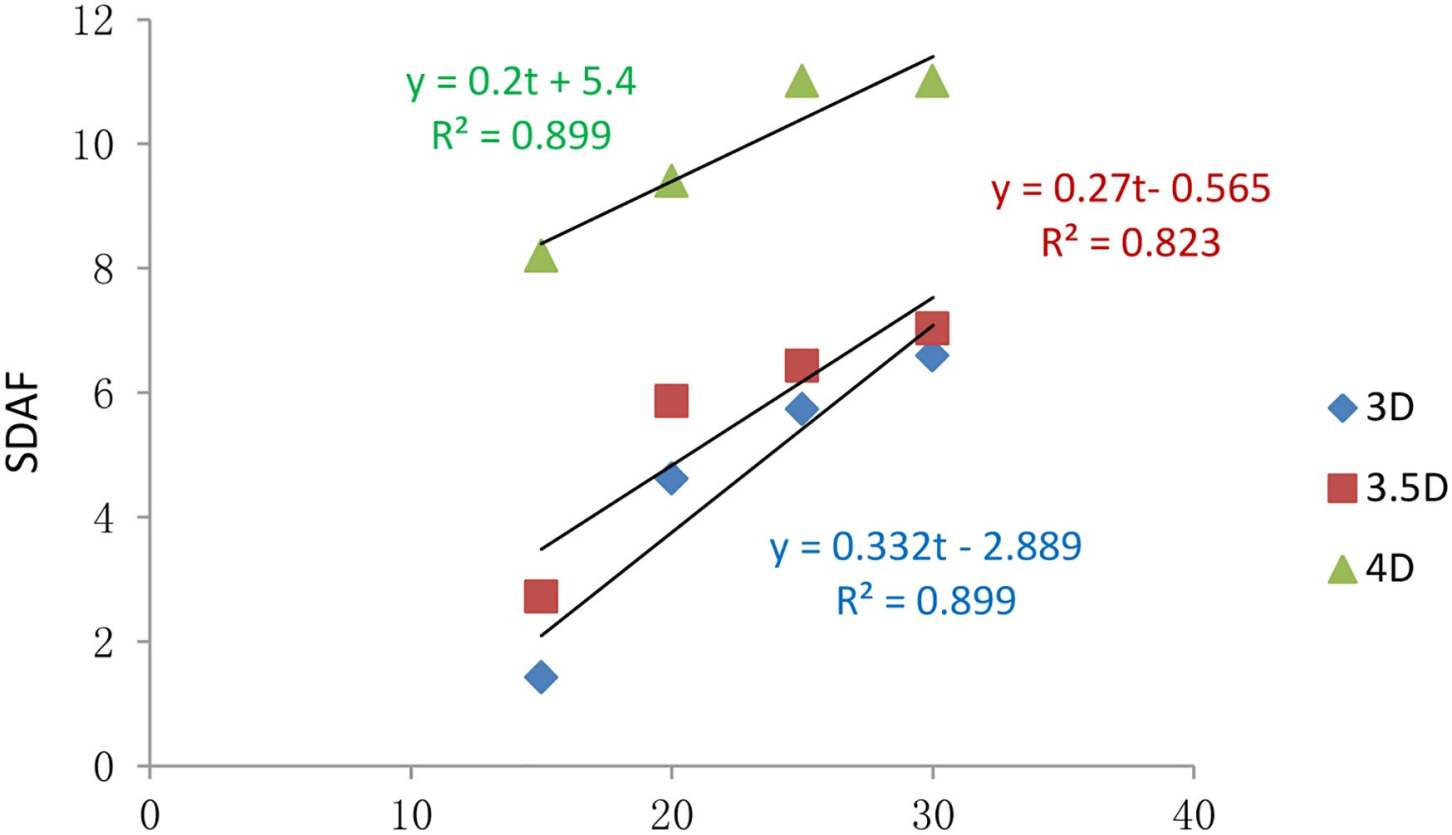
Changes in SDAFS with vibration time for the different spacing conditions.

The change curves of the SDAF with vibration time under different conditions were linearly fitted, and the results are shown in Fig 14.

The linear relationship varied with the vibration time and working condition. Specifically:

When the pile spacing was 3D, *y* = 0.332*t* − 2.889, and *R*^2^ = 0.899.
When the pile spacing was 3.5D, *y* = 0.27*t* + 0.565, and *R*^2^ = 0.823.
When the pile spacing was 4D, *y* = 0.2*t* + 5.4, and *R*^2^ = 0.899,

where y is SDAF, t is time (s), and *R*^2^ is the coefficient of correlation.

According to Fig 14, the SDAF was the largest under the 4D conditions, followed, in decreasing order, by the 3.5D and 3D conditions at the same vibration time. The SDAF increased with increasing pile spacing, which demonstrated that the vertical bearing capacity of the composite pile foundation system of the 3D pile spacing was the highest.

In conclusion, a longer vibration time leads to higher seismic energy, after which the settlement and SDAF increase.

Through the calculation and analysis of the SDAF, the static load can be multiplied by the SDAF calculated above so that the dynamic problem is transformed into static analysis, with the result forming the basis for the dynamic design of pile foundations.

## 3 Conclusions

The dynamic characteristics of pile group systems were studied using a small shaking table test with a 3 × 3 pile group structure. Based on the time history analyses of the excess pore pressure ratios and vertical settlements under the four study conditions, the following conclusions are drawn:

1. The low cap pile group system can improve the anti-liquefaction performance of the soil. The degree of improvement is related to the pile spacing. Based on the test, the 3D conditions provided the highest anti-liquefaction performance, followed, in decreasing order, by the 3.5D and 4D conditions. These results should provide a basis for the proper selection of pile spacing in the seismic design of a pile foundation.
2. In the vibration process, the time history of the settlement can indirectly reflect the change in the vertical bearing capacity of the pile foundation. The SDAF was introduced, and the linear relationship between the vibration time and pile spacing was fitted. The SDAF was calculated based on this linear relationship and then multiplied by the static load, resulting in the basis for the dynamic design of the pile foundation. Thus, the dynamic problem was transformed into static analysis, making the dynamic calculation of the pile foundation simple.

## Supporting information

S1 Data (RAR)
